# Supplementary material for: Profiles of competence development in upper secondary education and their predictors
Source: PLoS One. 2021 Jan 29;16(1):e0245884. doi: 10.1371/journal.pone.0245884 (PMC7845973; doi:10.1371/journal.pone.0245884)
Supplement: Attachment — Submitted filename: Response to reviewers.docx [file pone.0245884.s001.docx]

Dear Dr. Kovanovic, dear reviewers,

together with this rebuttal letter, you may find the revised version of our manuscript entitled “Profiles of competence development in upper secondary education and their predictors”. We thank the reviewers for their thoughtful and helpful comments. The specific changes made regarding the reviewer comments are detailed in the tables below.

Comments and changes in response to Reviewer #1

| **Comments of the reviewer** | **Comments and changes to the manuscript** |
| --- | --- |
| Section 3.3  I would recommend including a table of the data (if possible) such as listing attribute names and values that will feed your model. This would allow the reader to gain a better understanding of what data you included and the relationship it has with your hypothesis and methodological approach. | We want to thank the reviewer for this recommendation and agree that descriptive information of the data and the connection of the data with our analyses were missing.  To introduce both types of information to the reader, we included Table 1 (in 3.3) and Figure 1 (in 3.4). Table 1 offers an overview of the variables, grouped by their later usage, with information on transformations that were necessary to prepare the variables from the original dataset for our analyses and information on the range of values. Figure 1 adds a summary of the datasets used for all methodological procedures (i.e., imputation, latent growth mixture modeling and multinomial regression) including the variables use for these procedures.  Overall, we believe that these additions provide a clarification regarding the variables and planned procedures. |
| Section 3.4.3 Include a table (if possible) that shows the processes of model evaluation, i.e. best fit statistics, number of classes and associated model. This will help the reader how you determined which model was the best choice. | We followed this advice and included Table 2 (section 3.4.3) that provides an overview for readers regarding the process of model selection. |
| My only concern is how do you know for sure that a generalised and a specialised profile of competency will be the only viable latent profiles? What if the model yields a competency based profile that is completely unexpected? For example, a latent profile that shares both characteristics of a generalised and specialised competency or perhaps something entirely different? How would you address a model that recommends having 4 or 5 latent profiles as the best fit? It did not seem clear to me that the data used and methodology can and only yield either a generalised or specialised profile of competency. | We appreciate this comment because it addresses a critical point in the protocol. We made several changes with the aim of clarifying the process of profile identification, profile interpretation, and profile differentiation. While the reasoning behind the three profiles of competence development might seem obvious to us, this comment highlighted a potential lack of clarity in our presentation that lead to multiple changes in several sections:  - Section 3.4.1: We added the word ‘linear’, to indicate, that only intercept and slopes are relevant for latent growth in our model (with only the slopes being relevant for the identification of profiles).  - Section 3.4.4: We added an explanation that the model based on the two slopes can only result in specialized profiles in either domain or generalized profiles.  - Section 3.4.4: We added the concept of “profile types” in the section and included a new paragraph on the implications of more than 3 profiles. After identification of the most appropriate profile configuration, the profiles will be grouped into types to examine the hypothesized predictors.  Overall, we are very thankful for this comment regarding the possible profiles. This comment offered us the opportunity to revise the original wording and, hopefully, clarifying the idea of the different types of profiles. |
| Regardless, I believe this research has a lot of potential. | We very much want to thank the reviewer for these kind words. |

Comment in response to Reviewer #2

| **Comments of the reviewer** | **Comments and changes to the manuscript** |
| --- | --- |
| This seems like a straightforward study for which the origin of the data, as well as the purpose is very clear. Maybe the discussion and analysis of all presented background variables in relation with the latent profiles is a bit too ambitious but I nevertheless deem them feasible. I'm looking very much forward to the results. | We thank the reviewer for the positive evaluation. |

In addition to the above mentioned changes, we made some minor additional changes and clarifications to improve the quality of the manuscript.

We hope the paper is revised satisfactorily in view of the reviewers’ detailed and thoughtful comments. We really appreciate your and the reviewers’ helpful feedback and effort. With the hopes that the present version is now acceptable for publication in PLOS ONE we remain

Sincerely,
Micha-Josia Freund, Ilka Wolter, Kathrin Lockl, & Timo Gnambs
